# Posterior Pole Sparing Laser Photocoagulation Combined with Intravitreal Bevacizumab Injection in Posterior Retinopathy of Prematurity

**DOI:** 10.1155/2014/257286

**Published:** 2014-12-28

**Authors:** Rebecca Kim, Yu Cheol Kim

**Affiliations:** Department of Ophthalmology, Keimyung University School of Medicine, Dongsan Medical Center, 56 Dalseong-ro, Jung-gu, Daegu 700-712, Republic of Korea

## Abstract

*Purpose*. To report the results of the posterior pole sparing laser photocoagulation combined with intravitreal bevacizumab injection (IVB) in retinopathy of prematurity (ROP). *Methods*. A retrospective chart review of premature babies with ROP, all of whom received laser photocoagulation with IVB. Eleven eyes of 6 infants with advanced zone I ROP underwent laser ablation sparing posterior pole with concurrent IVB. The results were compared with those of full-laser treatment combined with IVB to 8 eyes of 5 infants with advanced ROP without involvement of the posterior pole. *Results*. The posterior pole sparing laser with IVB was performed with zone I, stage 3+ ROP at the mean postmenstrual age of 36 weeks and 5 days. The plus sign decreased significantly at postoperative day 1, the neovascular proliferation regressed by postoperative week 1, and the normal vascularization started at postoperative day 32 on the average. Two months after treatment, vascularization of the spared avascular area was completed. There was no macular dragging, tractional retinal detachment, foveal destruction by laser scars, or any other adverse event. No significant anatomical differences were identified from those of full-laser ablation combined with IVB. *Conclusions*. Posterior pole sparing laser with IVB can give favorable results without destruction of posterior pole retina.

## 1. Introduction

A current standard therapy in the treatment of advanced retinopathy of prematurity (ROP) is to ablate the peripheral avascular area by making laser photocoagulation scars without skip areas [1–3]. However, zone I ROP had approximately 55.2% unfavorable structural outcome rate in Early Treatment of Retinopathy of Prematurity (ETROP) trial, although conventional laser therapy was applied at optimal timing [4].

Retinal vascular complexes grow from the optic disc towards the periphery and, therefore, the further the retinal area from the optic disc is, the greater the likelihood it will remain avascular will be. However, the vascularization is possibly not equal in all directions in either onset or speed, which may contribute to the development of temporal avascular notch. One hypothesis could be that the development courses of the temporal retinal vessels are not straight lines, but arcades, and the temporal periphery of 3 or 9 o'clock may be the latest vascularized portion, which may explain why avascular areas protrude towards posterior pole at temporal median raphe [5].

**Figure 1:**
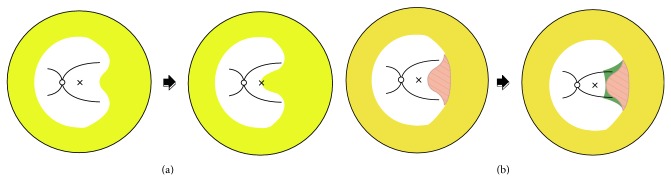
Diagrammatic presentation of the dilemma in the treatment of APROP. (a) Confluent laser ablation (yellow) without skipped areas; the expansion of the scar may induce the cecocentral scotoma. (b) Posterior pole sparing laser to avoid the scotoma; the spared area (red slash) can induce the fibrovascular membrane (green), as well as macular traction and distortion. APROP: aggressive posterior retinopathy of prematurity.

Advanced posterior ROP with the temporal avascular notch too close to the macula poses a dilemma for treatment. Conventional laser therapy including an avascular area near the fovea may cause a visual field defect (Figure 1(a)) [6]. On the other hand, laser ablation sparing the avascular area adjacent to the macula may allow extraretinal fibrovascular proliferation in the skip area, possibly inducing tractional membrane or macular dragging (Figure 1(b)).

To resolve this dilemma, the authors report the results of the posterior pole sparing laser ablation combined with intravitreal bevacizumab injection (IVB), which halts neovascularization immediately and preserves the macula from laser scarring by sparing the temporal avascular area close to fovea.

## 2. Materials and Methods

This study was approved by the Keimyung University Institutional Review Board. A consecutive retrospective review of all medical records of the infants who were treated with laser photocoagulation and IVB at our hospital for the treatment of zone I ROP or aggressive posterior zone II ROP from January 2006 to January 2012 was performed [7].

**Figure 2:**
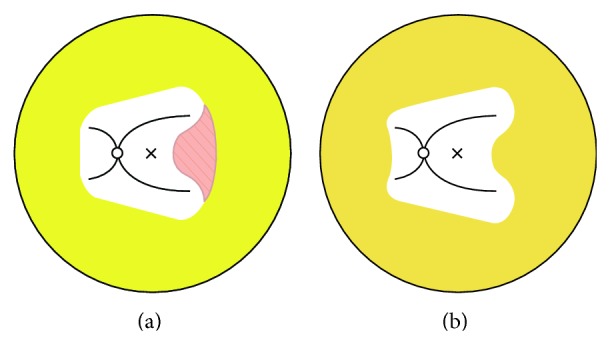
Diagrammatic presentation of the two groups. (a) In Group 1, with avascular area within 2 DD from foveal center, laser therapies sparing posterior pole with IVB were applied. (b) In Group 2, without avascular area within 2 DD from foveal center, complete confluent laser without spared zone and IVB were performed. DD: disc diameter; IVB: intravitreal bevacizumab injection.

In the ROP cases with avascular area within 2-disc diameter (DD) from foveal center, laser therapies sparing posterior pole with IVB were applied, defined as Group 1 (Figure 2(a)). In the eyes with ROP without avascular area within 2 DD from the foveal center, IVB and laser without the spared zone were performed, defined as Group 2 (Figure 2(b)).

Prior to the procedures, informed consent regarding the off-label use of bevacizumab (Avastin; Genentech, Inc., San Francisco, CA, USA) was obtained. All the bevacizumab injections were prepared from new Avastin vials just before the procedures on the day. Under general anesthesia, a near confluent pattern of laser photocoagulation was applied to the entire avascular retina except spared area, using laser indirect ophthalmoscope (VISULAS 532s, Carl Zeiss Meditec, Dublin, CA, USA). After bilateral laser treatments, 0.5 mg (0.02 cc) bevacizumab was injected temporally approximately 1.5 mm posterior to the limbus using a 30-gauge needle following disinfection with 5% povidone iodine. The perfusion of optic nerve head was then confirmed with indirect ophthalmoscope and paracentesis was performed when the intraocular pressure was high. Cravit ophthalmic solution (levofloxacin 0.5%, Santen, Osaka, Japan) and Cyclogyl 1% ophthalmic solution (cyclopentolate 1%, Alcon, Fort Worth, TX, USA) were prescribed to begin immediately and be continued every 6 hours and every 8 hours, respectively, for 6 days after IVBs.

The authors monitored ocular or systemic adverse events associated with drug or procedure and evaluated the courses of ROP with indirect ophthalmoscope during followup. The macular dragging was defined as ectopic or folded fovea. Statistical analysis used SPSS version 18.0 (SPSS, Inc., an IBM Company, Chicago, IL, USA). The Mann-Whitney *U* test was used for comparison of the two groups. Null hypotheses of no difference were rejected if *P* values were less than 0.05.

## 3. Results

Eleven eyes of six patients received posterior pole spared laser photocoagulation with the IVB (Group 1), and 8 eyes of 5 patients were treated with conventional laser treatment with the IVB (Group 2). In one patient, the left eye was in Group 1 and the right eye was in Group 2. In Group 2, one patient received an IVB only in the left eye.

The demographics of patients are presented in Table 1. The mean gestational ages at birth were 28.7 weeks in Group 1 and 27.1 weeks in Group 2. Mean birth weight was 1295.5 g in Group 1 and 1012.5 g in Group 2. The eyes involved in Group 1 are all stage 3 and zone I. There were no significant differences in birth weight or gestational age between the two groups. The average follow-up periods were 27 months (16–42 months) in Group 1 and 21 months (16–27 months) in Group 2.

The mean postmenstrual age (PMA) at treatment was 36.7 weeks in Group 1 and 36.3 weeks in Group 2. The plus signs started decreasing significantly at postoperative day 1 in both groups and disappeared after 2.1 days after the treatment in Group 1 and after 1.8 days in Group 2. Normal vascularization was noted to have started at postoperative day 32 on the average and to have been completed over the laser spared area about 2 months after the treatment (Group 1: 54.8 days; Group 2: 57.2 days), and peripheral retinal vessel development on some adjacent laser scar area was then observed. Through the last followup, no vision-threatening laser scar expanding to the fovea was observed. There were no significant differences in the resolution of the plus sign and in the duration of peripheral vessel growth up to the laser scar between the two groups (Table 2).

Group 1 received 2631.8 of laser burn during the procedure, and there is an average of 218 laser spots less compared to Group 2. The difference of the spot counts is presumed to come from the sparing areas.

Both groups were observed to have complete regression of the ROP without recurrence of fibrovascular membrane, macular dragging, or retinal detachment, and other serious ocular complications and any other systemic complications are not identified.

## 4. Discussion

Following Campbell's description [8] of the relationship of ROP to oxygen exposure, cryotherapy for retinopathy of prematurity (Cryo-ROP) [9] study and ETROP trial have contributed to the development of ROP treatment. However, the ETROP study reported over 50% unfavorable structural results in zone I disease [2, 3]. Although laser photocoagulation has permanent effect on regression of neovascularization, by destructing avascular retina with overproduction of vascular endothelial growth factor (VEGF), it may increase VEGF temporarily immediately after photocoagulation without effect on VEGF already present in the vitreous cavity [10, 11]. For that reason, it takes 2-3 weeks for regression of neovascularization and ROP can progress, especially in posterior pole ROP (zone I or posterior zone II ROP) despite full-laser treatment. Besides, laser scar in temporal retina may creep and enlarge to macula afterwards, resulting in scotoma or central vision defect, as reported in diabetic patients with panretinal photocoagulation, although it has not been documented in ROP literature [12].

Although intravitreal bevacizumab does not act over a long period, it immediately starts inducing regression of the neovascularization by blocking all VEGF in the vitreous [6, 11]. For that reason, anti-VEGF therapy has an additional benefit over laser therapy; furthermore, prior studies have demonstrated IVB as an adjuvant or as a possible monotherapy [6, 13, 14]. This study obtained good results by applying posterior pole sparing laser with IVB to posterior pole ROPs, instead of the standard treatment of ROP. The results are assumed to come from the complementary cooperation of laser therapy with late-onset but long-term effects and IVB with early onset but short acting.

Initially, anti-VEGF decreases VEGF and halts the advance of the neovascularization immediately and then the laser effects begin in 2-3 weeks [11]. The effects of anti-VEGF dissipate in 2 months, but it is assumed that the amount of VEGF from the spared avascular retina is too little to induce extraretinal fibrovascular proliferation. Subsequently, normal retinal vascularizations are completed. This current study also showed that complete retinal vascularization was identified in 2 months after treatment. Additionally, the vascularization on the laser scar was observed, although the Bevacizumab Eliminates the Angiogenic Threat of Retinopathy of Prematurity (BEAT-ROP) study stated that permanently destructed peripheral retina by conventional laser treatment does not allow for normal retinal vascularization [15]. Some study [16] also showed peripheral retinal vascularization on the laser scar in the ROP cases with laser treatments and IVBs, which implies that it is possible that IVB is helpful for peripheral retinal vascularization on laser scar by inducing complete regression.

When IVB was first applied to the treatment of ROPs, it was used as an adjuvant to laser [6, 13]. It was thought that permanently effective laser would compensate for short-term effective bevacizumab. However, since bevacizumab monotherapy for the ROP cases in which laser therapy was impossible because of poor retinal visualization showed good results [17], the following reports of successful monotherapy for advanced ROPs have proposed IVB as an alternative to laser rather than as an adjuvant.

There are several possible explanations about why repeated IVBs are not necessary in ROP, unlike other retinal diseases that necessitate repeated injections. As the PMA increases, especially over the age of 45 weeks, the infant is at lower risk of progression of ROP [14, 18, 19]. Anti-VEGF can suppress ROPs definitely during the critical period. After exhaustion of the anti-VEGF, the VEGF concentration is presumed to increase gradually to the proper level to induce normal retinal vascularization.

The BEAT-ROP trial [15] compared intravitreal bevacizumab (0.625 mg in 0.025 ml) monotherapy and conventional laser therapy in zone I and zone II ROPs and concluded that IVB monotherapy is superior to laser for treatment of zone I, stage 3+ ROP. Nevertheless, two recurrent cases 19.2 ± 8.6 weeks after IVB were reported in zone I ROP. This likely resulted from termination of the medicinal effect rather than from lack of the anti-VEGF potency, comparing the interval (6.4 ± 6.7 weeks) from laser to recurrence [20, 21]. A similar recurrence period gap between IVB and laser was observed in zone II ROP too. Hu et al. [22] also reported 9 infants (17 eyes) with recurrence of ROP after initial treatment with IVB monotherapy and concluded that, although IVB treatment is effective in inducing regression of ROP, the effect may be transient and late retinal detachment can occur despite early regression. It implies that about 5% of advanced zone I or II ROPs may produce excessive VEGF to induce neovascularization, after cessation of anti-VEGF effect. On the one hand, bevacizumab monotherapy may be a simpler and more effective treatment than laser therapy, but it still has a 5-6% recurrence rate. Combination treatment can resolve the recurrence problem. In the current study, there was no recurrent ROP case after combination treatment, during the follow-up period (16–42 months), for longer than the recurrence intervals reported in IVB monotherapy. Besides, combination with laser can be expected to prevent retinal detachment complicated by IVB [23].

Repeated injections for the treatment of ROP, as in other neovascular retinal diseases, may be another option. However, this may be dangerous, considering systemic adverse effects of anti-VEGF. Sato et al. [24] reported that the serum VEGF levels dropped 2 weeks after IVB 0.5 mg.

Although IVB monotherapy as the initial intervention and close observation for laser treatment as needed is also possible, the recurrence period after IVB is too long for close observation. Hu et al. [22] reported that the mean time between initial IVB and treatment-requiring recurrence was 14.4 weeks, with a minimum of 4 and a maximum of 35 weeks, and recurrence after IVB can occur later than with conventional laser therapy. Close observation over 7 months is required after IVB, unlike laser photocoagulation, because timely laser therapy is essential for the treatment of recurrent ROP [25]. If the patient is at risk for leaving observation or needs to do a single treatment session due to anesthesia or other non-retina reasons, this would be a good treatment option.

When anti-VEGF was first applied to the treatment of ROPs, IVB was performed after laser treatment, because with the uncertain effect on ROP at that time clinicians were reluctant to use bevacizumab as an initial treatment. However, IVB given during the fibrovascular organization following laser for ROP may induce acute contraction of the proliferative membrane and aggravate tractional retinal detachment. Accordingly, intravitreal bevacizumab should be injected before laser or at the same time [23, 26].

Recently, Kim et al. [27] reported that 18 eyes with type 1 ROP in zone I underwent combined zone I sparing laser with IVB. All eyes showed prompt regression of neovascular pathology and plus disease. Vascularization reached zone II without recurrence or adverse events after the treatment. However, the current study spared posterior pole and compared the results with those of full-laser treatment combined with IVB. Sparing posterior pole of which center is fovea is presumed to conserve better visual function than zone I.

Even though the combination treatment of laser and IVB has 100% success rate in ROP regression in this series of patients, it also should be reserved only for APROP or some severe posterior ROPs, because most of ROPs can be successfully treated with IVB alone or laser monotherapy, and there is potential risk in that blood retinal barrier might be affected by laser [28] and anti-VEGF might leak into the systemic circulation more than the IVB monotherapy, which could increase systemic effect and reduce local effect of anti-VEGF.

This study is limited by its retrospective design, with a small number of ROP cases; the follow-up intervals were not tight enough to check all the changes of the ROP courses; the follow-up periods were not long enough to check the visual field defect and the refraction of the eyes; the nonvascularized zones temporal to fovea of the two groups were different, although the other basic characteristics are not significant. However, the less vascularized Group 1 ROP had favorable results, even with less invasive and destructive laser treatment. Additionally, the safe dose of IVB for ROP infants has not been proved yet and a half dose for adults could be harmful systemically for premature infants, especially after laser treatment. The patients need to be followed continuously to claim the real benefits such as less central scotoma on the visual field and to check possible side effects through additional functional tests.

In conclusion, in zone I or posterior zone II ROP in which laser treatment alone may be dangerous, posterior pole sparing laser with IVB can induce favorable results without destructing the posterior pole retina, compared with the conventional ROP treatment and other IVB treatments. Further studies are needed to establish how much avascular retina can be spared in combination treatments and which ROP cases need IVBs.

## Figures and Tables

**Table 1:** Demographics of infants in Group 1 and Group 2.

	Group 1	Group 2	*P* value^1^
Birth weight (g)	1295.5 ± 290.0	1012.5 ± 85.3	0.08
Gestational age (weeks)	28.7 ± 1.3	27.1 ± 0.7	0.08
Gender (M : F)	5 : 1	2 : 3	
Delivery (C-sec : VD)	7 : 4	8 : 0	
Stage	All 3	All 3	
Zone 1 : zone 2	All zone 1	2 : 3	

C-sec: caesarean section; VD: vaginal delivery; ^1^Mann-Whitney *U* test.

**Table 2:** Comparison between Group 1 and Group 2.

	Group 1	Group 2	*P* value^1^
Mean age at treatment (postmenstrual age, weeks)	36.7	36.3	0.840
Loss of plus sign after treatment (days ± SD)	2.1 ± 1.16	1.8 ± 0.9	0.657
Peripheral vessel growth after treatment (day ± SD)	54.8 ± 18.4	57.2 ± 6.4	0.177
Mean number of laser burns	2631.8	2849.3	0.717

^1^Mann-Whitney *U* test.
